# What evidence exists on the impact of climate change on real estate valuation? A systematic map protocol

**DOI:** 10.1186/s13750-023-00317-y

**Published:** 2023-11-18

**Authors:** Fedra Vanhuyse, Tommaso Piseddu, Åsa Moberg

**Affiliations:** 1 Stockholm Environment Institute Linnégatan 87D, Box 24218, 104 51 Stockholm, Sweden; 2 Department of Real Estate and Construction Management, KTH – Royal Institute of Technology, Stockholm, Sweden

**Keywords:** Natural disasters, Economic losses, Climate risk, Transition risk, Real estate

## Abstract

**Background:**

As natural disasters increase in both frequency and magnitude because of climate change, assets, such as buildings and infrastructure, are exposed to physical climate risk. In addition, as our societies transition towards a greener economy, the transitional climate risk will manifest itself in different forms: reputational issues, market solutions that may drive out those that do not comply, technological disruptions and policy initiatives. How both risks, physical and transitional, impact the economic value of real estate assets is not well understood and will be investigated as the main scope of this systematic map.

**Method:**

we use systematic mapping to collate and configure existing evidence on how climate risk has affected the economic value of real estate assets. After designing a search string, English language peer-reviewed publications will be retrieved from the two largest and most popular scientific research databases, as well as a database containing policy documents. This corpus will be tested for comprehensiveness using a benchmark of 50 highly relevant articles. Once the comprehensiveness test is passed, a consistency test will be carried out on the screening of a randomly selected list of 200 articles by three reviewers. If a kappa score of at least 0.6 is achieved, one of the reviewers will carry out the remainder of the screening, with another reviewer quality assuring 10% of the screening. The retained corpus will then be distributed over the three reviewers, who will carry out the extraction of metadata according to an agreed coding strategy. The final output of the coding will consist of a heat map, showcasing where substantial evidence is available, and research gaps, providing recommendations for further research. In addition, the results will provide insight into the methodology to quantify the impact of climate risk on real estate value. Figures and tables will be designed to make it easy to comprehend the results of the mapping.

**Supplementary Information:**

The online version contains supplementary material available at 10.1186/s13750-023-00317-y.

## Background

The increasing number of extreme weather events and their adverse impacts on economies have triggered new concerns by financial authorities and investors around the world. The Global Risks Report 2023 [1] lists 5 environmental-related risks among the 10 most severe risks for the global economies in the coming years, including “Failure to mitigate climate change”, “Failure of climate-change adaptation” and “Natural disasters and extreme weather events” among others. Climate risks can be either physical or transitional [2] and these risks impact on banks, and financial institutions, either directly through the valuation of assets, liabilities and cost of capital, lower corporate profitability, or indirectly, through macro-financial changes.

To understand climate risks, several financial institutions, such as the European Central Bank, and the Swedish National Supervisory Authority Riksbanken, have drafted regulations and produced guidelines for financial actors related to the incorporation of climate risks throughout their operations [2, 3]. However, most financial market actors do not sufficiently assess, or report on, the climate change risks, which poses a substantial liability [4]. The ECB found that none of the 109 lenders it supervises meet its climate disclosure expectations [5]. Only a minority of the Principles for Responsible Investment (PRI)’s signatories uses scenario analysis to estimate the impacts of climate change on the value of their investment portfolio [6]. Less than half the organisations interviewed by The Global Association of Risk Professionals (35 of the 78 banks, asset managers, and insurers) used climate scenario analysis on their portfolios in 2021 [7]. In addition, research shows substantial inconsistencies between the physical climate risks used by investors [8].

The size of the real estate market suggests that the exposure to climate risks may generate substantial negative consequences to the national economies. The total assets value of some European real estate companies is as large as €60 billion and this makes some of these companies among the largest in the world by market value [9]. The presence of these companies on the stock markets determines a condition where investors, either private or institutional, are exposed to financial climate risks too through the purchase of financial products by these companies. In some countries, as many as 1 out of 10 real estate companies are listed on the national stock markets [10]. If the impacts of climate change, both in the physical and in the transitional climate risk form, were to be incorporated into the values of the assets that these companies hold, some financial systems would be highly exposed. Larger impacts at the macro-financial level may then be expected as a result of such an exposure.

As climate risks and finance have emerged only recently as a political priority [11], the scientific body of knowledge linking both is, understandably, not well developed. Whereas some actors in Sweden such as Handelsbanken [12] have published an analysis, no academic research on climate risk and material asset valuation in Sweden was found. A recent publication from [13] summarized the research challenges in evaluating the economic risks of climate change to include: (1) understanding the heterogeneity of agents, their risk preferences and vulnerability; (2) simultaneous and cascading impacts; and (3) regional heterogeneity. Understanding the methods that integrate climate risk into the value of financial assets, and real estate assets in particular, seems highly pertinent [14–16].

### Stakeholders’ engagement

With this systematic map, we aim to identify trends and gaps in literature around the effects of exposure to climate risk on the value of real estate assets and inform a case study in Sweden, responding to a call to “systematically evaluate risks under alternative scenarios of future climatic and societal conditions” [17]. The relevance and the results of our research are tested through constant engagement with the industry partners that are participating in the Vinnova-funded MAVERIC project, which aims at understanding how climate risks materialize in real estate valuation methods. Our partners represent different types of actors, including real estate owners, real estate evaluation companies, a financial institution, a government agency, and universities, all active in Sweden. This diverse perspective of our partners allows us to consider all possible impacts and methods therefore guaranteeing a holistic view of the topic.

### Objectives

This systematic map is intended to understand the main themes in the research literature around how climate risks affect the economic evaluation of real estate assets. While there is no standardized way to classify real estate assets, most often these are grouped into three main sectors: (1) residential assets such as houses, apartments, villas, or other buildings that mainly serve for housing and non-professional purposes; (2) commercial real estate, including venues such as offices, hotels, shopping malls and, more in general, any space that is used for business and professional purposes and retail activities; and (3) industrial real estate, that comprehend venues that are used to host one or more phases of production activities or to provide services, such as warehouses and factories and assets leased to the public sector. For this map, we use [18]: residential, offices, industrial, retail, hotels, and others. Climate risks can be categorized as physical risks or transition risks [19, 20]:

- **Physical risks** arise from climate change impacts and climate-related hazards, including risk to facilities and infrastructure, impact on operations and resource availability. They can be acute short-term events or chronic long-term changes. Physical damages usually take two forms: structural and non-structural damages. Structural damages represent those cases where a structural modification is produced and as a result of that we observe a deterioration of the physical properties and behavior of the building [21]. Examples of structural damages can range from the complete destruction of the building to holes in roofs and walls**.** Examples of non-structural damages include, for instance, damages to ornamental features of the buildings, the collapse of a balcony balustrade or the displacement of roof tiles.
- **Transition risks** are related to the transition to a low-carbon economy and include policy and legal risks, liability risk, technology risk, market risk, and reputation risk. Policy and legal risk include the effects of policy action to mitigate climate change and action to promote adaptation to climate change. Policy risks include e.g., increased taxation or costs related to energy performance or adaptation requirements. Legal risks are also related to climate-related litigation claims, due to failure to mitigate or adapt to climate change and to insufficient disclosure of risks. Technology risk covers the improvements and innovations needed for the transition, which can lead to increased costs and changes in competitiveness. Market risk covers changes in supply and demand for certain products and services as climate-related aspects are considered. Reputation risk relates to the perceptions of customers and community.

Natural hazards are here considered as in the definition provided by [22]: extreme events, of natural origin, that carry the potential to generate damages both to societies and to individuals. They can be organized into hydrometeorological events (storms, extreme temperature events, forest fires, water scarcity and droughts and floods) and geophysical hazards (avalanches, landslides, earthquakes and volcanic eruptions) [23]. The EU Taxonomy, however, does not include earthquakes and volcanic eruptions in the classification of climate-related hazards. Instead, risks are classified as temperature-related, wind-related, water-related or solid mass-related, and can be acute or chronic [24].

**Fig. 1:**
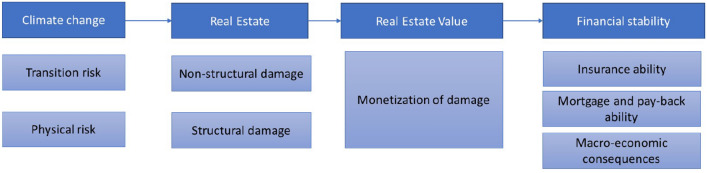
Overview impact pathway

The impact pathway underpinning our research questions is visualized in Fig. 1- noting that the consequences for financial stability are out of scope for the systematic map.

### Primary questions

Given the objectives described above, our systematic map aims to answer the following research questions:

- RQ1. How have climate risks for real estate markets been described in the literature?
- RQ2. How have climate risks been seen to impact real estate, and in particular real estate values?

### Components of the primary questions (PECO)

Based on the two research questions outlined above, the (Population, Exposure, Comparison, Outcome) structure can be used to construct as follows:

- Population: real estate assets (Offices, Industrial and Logistics, Retail, Residential, Healthcare, Hotels, etc.) Geographically, we filter to Europe and North America (USA and Canada).
- Exposure: exposure to climate risks, whether physical climate risks or transitional climate risks, actual, based on a model, on future projections or on valid assumptions
- Comparison: pre- and post-comparisons with the value of the asset, comparable assets with similar characteristics in the proximity that are not exposed to the risks, value of the asset in the absence of exposure to risks
- Outcome: a variation in the value of the assets that are subject to any form of climate risk; an estimate in absolute monetary units, monetary units per m^2^, or a percentage variation

### Secondary questions

A set of secondary questions guide the review of the corpus in replying to the primary questions:

- What is the typology of natural hazards considered in the literature? Are certain types of hazards more commonly investigated?
- How are transitional climate risks considered in the literature?
- What are the countries that received most attention in previous studies? Is there any correlation with the high exposure of these countries to certain events in the past?
- How are actual damages and potential risks accounted for in economic terms? Do researchers specifically focus on one or more of the aspects of real estate valuation methodologies?
- What are the categories of real estate assets that were subject to particular attention by previous research?

### Methods

We selected a systematic map as the preferred methodology over a systematic review, stemming from the fact that this is an emerging research theme and that we do not aim to quantitatively address our research questions [25]. Instead, we collect evidence on the topic and identify trends in the literature and potential gaps.

**Fig. 2:**
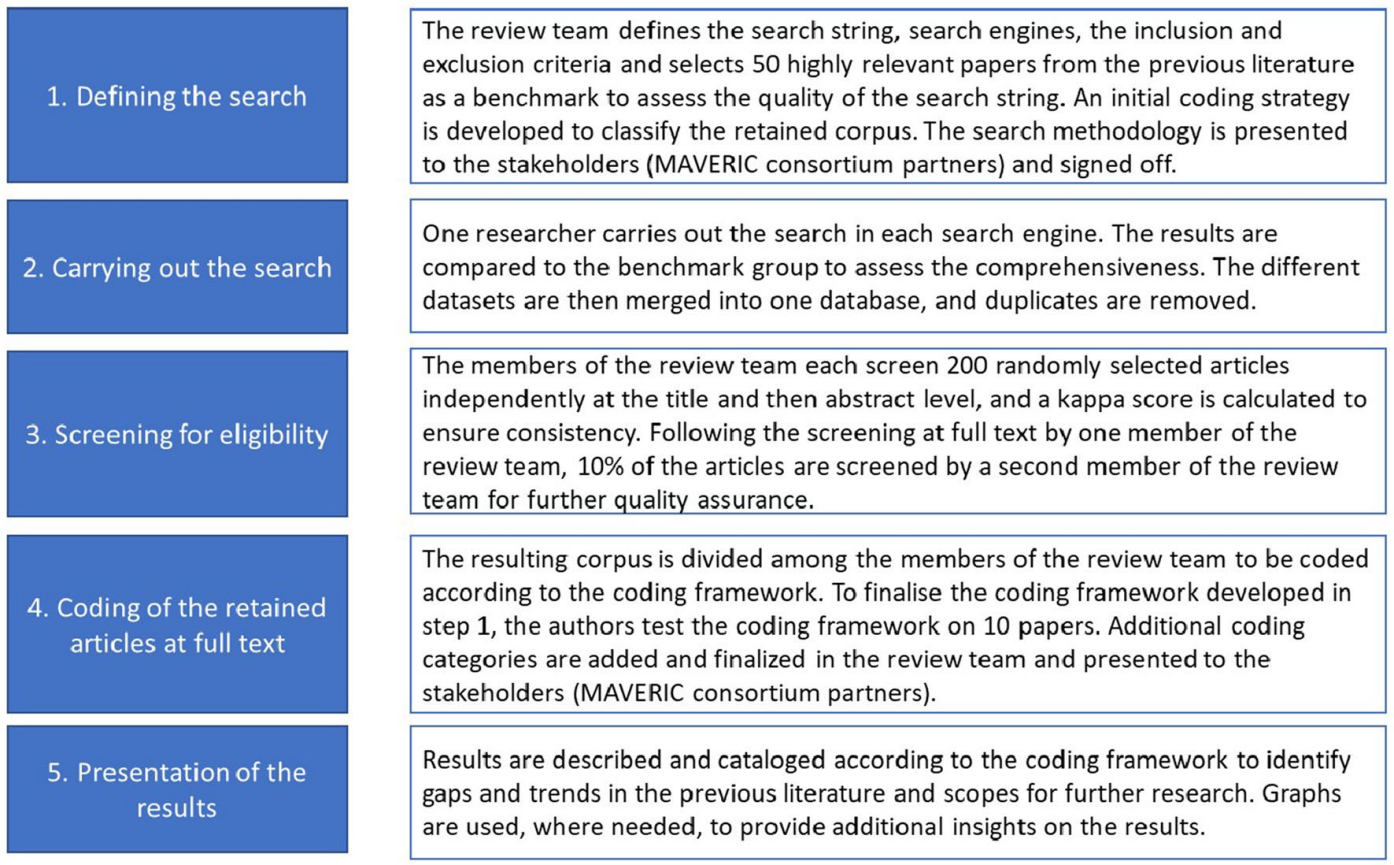
Overview research process

We follow the approach that has been developed under the PRISMA framework [26], the standard for reviewing existing knowledge around a certain theme. The rest of this protocol is structured around the main points of this framework with the additional actions to guarantee a rigorous and replicable systematic map. Figure 2 contains the overview of our research process.

### Searching for articles

#### Search string and language

Our search string comprises different thematic topic areas, each of which will contribute to responding to our research questions. The first topic area is the one revolving around climate risks. The second topic area deals with how these could impact real estate, i.e., the economic or the financial evaluation of the assets at risk. The third and last topic area of the search string is the one that aims at capturing the object of our review, real estate assets. Its construction is based on the elements of the PECO framework that we outlined above. To ensure we capture as wide a set of research, we apply these search terms at all levels (title, abstract, keywords and full text).

The resulting search string is therefore:

ALL (climat* AND risk* AND (value OR economic OR financ*) AND (real AND estate OR building*)) AND (LIMIT-TO (DOCTYPE, "ar")) AND (LIMIT-TO (PUBYEAR, 2014) OR LIMIT-TO (PUBYEAR, 2015) OR LIMIT-TO (PUBYEAR, 2016) OR LIMIT-TO (PUBYEAR, 2017) OR LIMIT-TO (PUBYEAR, 2018) OR LIMIT-TO (PUBYEAR, 2019) OR LIMIT-TO (PUBYEAR, 2020) OR LIMIT-TO (PUBYEAR, 2021) OR LIMIT-TO (PUBYEAR, 2022) OR LIMIT-TO (PUBYEAR, 2023))

The search string presented above is the one for the search in Scopus. The search strings for the other search engines will look different as we need to account for different terms and search structures, but its components will remain unaffected. In Additional file 1, we provide the results from our search string using the different databases. Due to the large number of articles, we restrict the search to research published in the last ten years, i.e., since 2014. In addition, we restrict our search to similar geographies as Sweden, i.e., Europe and North America. Given language barriers, only research articles in English will be included in the final corpus.

#### Publication databases

Our Publication databases include Scopus (Elsevier), Web of Science and Overton. The selection of the academic datasets is based on previous literature, which identified them as the two leading databases in systematic mapping and systematic reviewing [27]; on considerations of the features that they present when compared to other alternatives such as Google Scholar, including, among many, the possibility to use complex search string with no limitations and user-friendly download and export options [28]. Finally, we made the decision to turn to these datasets on account of the high quality and rigorous content selection that has been recognized through the examples of previous uses in the literature [29]. We added Overton as a publication database as well. According to their website, Overton “is the world’s largest searchable index of policy documents, guidelines, think tank publications and working papers” [30]. In this database, we in particular aim to grasp grey literature and commentary papers on climate risk and real estate valuation methodologies, and possible impacts of climate change on real estate valuation. Our preliminary search (see Additional file 1) showed that the Overton search resulted in many papers. Therefore, we limit the number of included papers from Overton to 1000.

The results of our searches will be stored in two databases: one containing academic literature from the publication databases Scopus (Elsevier) and Web of Science; and one containing the Overton results. We do so to distinguish between peer-reviewed and non-peer-reviewed articles.

#### Internet searches

No Internet search will be conducted. Searches for grey literature such as working papers, opinion pieces, factsheets, policy briefs and reports will be conducted using Overton only. We note a potential lack of academic rigor, absence of a peer-review process, issues with data management, data extraction and replicability. The stakeholders of the MAVERIC project will be informed about the results of the searches and will be engaged in a discussion on the implications of the findings, but they will not be asked to contribute to the creation of the final corpus of documents.

#### Supplementary search

We acknowledge that supplementary methods could be used to guarantee that relevant papers that escape the search string are captured, such as the forward or backwards citations chasing. However, both on considerations of time constraints and confident that enough results will be returned by our proposed approach to provide a deep and meaningful systematic map, no citation chasing or any other supplementary search will be carried out.

#### Comprehensiveness of the research

A benchmark list of 50 relevant papers that comply with the criteria specified above (Additional file 1), with the inclusion criteria in Table 1 and that the reviewers deem relevant for the research has been constructed, and the corpus resulting from the search has been double checked to ensure that the search string is comprehensive enough to include these. About 75% of the articles are found among the resulting papers from the search string.

**Table 1:** Overview inclusion and exclusion criteria

Category	Inclusion	Exclusion
Population	Publication databases: Scopus, Web of Science and Overton	
	Objects of the analysis include: offices, residential assets, logistics assets, and other buildings. Locations of the studies include Europe and North America	Estimates of damage reported over other assets (land, land development projects, etc.) or other economic measures (GDP, industrial capacity, etc.). Case studies outside of Europe or North America
Intervention	Exposure to climate risks, both physical and transitional. Exposure can result from an actual event that involved the asset or from a potential risk assessment (e.g., risk maps produced by national authorities)	Estimates of assets’ values that do not include the impact from climate risk
Comparison	Assets’ values change can be accounted for in different forms, including but not limited to: • Pre- and post- value comparison • Comparison with other similar assets in the proximity • Resulting from a theoretical model • Estimate of damages	N/a
Outcomes	Variation in the asset value as resulting from the exposure to climate risks	No estimate of value change, whether from actual damage or from potential exposure to risk, is provided
Study type	Peer-reviewed articles from relevant academic journals, using the academic search engines Grey literature including reports, policy briefs, working papers, conference proceedings, conference papers, using the Overton search engine Publication period: after and including 2014 Published articles only	Conference proceedings, conference papers using the academic search engines Academic journals that have no clear or vague link with the research topic (e.g., medical journals) Before 2014 Unpublished articles and articles in press

### Article screening and study eligibility criteria

#### Screening process

Articles will be screened at the title, abstract and full text level for the academic article screening. One of the researchers in the team will screen the full corpus on title, abstract and full text level, and 10% of it will be screened by a second reviewer for quality assurance. Where unsure, articles will be included for review to ensure no articles were left out in error. A list of excluded articles will be produced and maintained together with the reasons that led the team to opt for their exclusions. For the Overton search results, articles will be screened at title and abstract level in a first instance, and then at full text by a researcher.

To ensure consistency in the screening process, a list of 200 papers will be randomly picked and screened independently at title and the abstract level by three researchers, after which the screening results will be compared. For consistency at the full text level, 20 papers will be randomly picked and screened independently. To assess the extent of agreement within the reviewing team, we will make use of a statistical measure to evaluate the robustness of the approach to potentially diverging screening standards. The kappa score introduced by [31] measures the degree of inter-rater reliability accounting for the fact that agreement may also occur by chance. Given the size of this reviewing group, we will adjust our approach following [32] which allows for the construction of a figure that considers multiple reviewers, solving the Cohen’s version limitation to only two reviewers. While this measure finds vast application in the literature, no agreement has been reached on the threshold that would define a solid process and different values are often observed [33–36]. Following the recommendation in [37], we set a kappa minimum score of 0.6. We will then compute the value of the kappa measure on the 200 articles that we randomly pick and screen at the title and abstract levels. If the test does not reach the required minimum kappa, the inclusion criteria will be discussed among the reviewing team and the criteria will be adjusted to reflect the results of this discussion. This screening procedure analysis and the computation of the kappa score are repeated until the minimum kappa threshold is reached, to ensure that screening criteria are well understood and consistently applied by all members of the reviewing team.

We pay special attention to making sure that no member of the review team is assigned a publication that she/he authored or co-authored. Should such a situation emerge, the publication will be assigned to another member of the review team. The results of the coding process are stored in a spreadsheet file format which is made available to every reader upon request.

#### Eligibility criteria

Table 1 contains our inclusion and exclusion criteria.

Journals whose title clearly indicates that the result is not relevant for the scope of our mapping will be excluded, including, for instance, “Frontiers in Sustainable Food Systems” and “International Journal of Hydrogen Energy” and medical journals. To avoid losing potentially relevant results we will only apply such a criterion if the evidence is clear and leave the article in the retained corpus when in doubt. Before carrying out our screening, we will seek approval from our stakeholders (the MAVERIC consortium members) on excluded journal titles. We will also include the overview of excluded journals in our systematic map methodology.

Geographical filtering to Europe and North America (USA and Canada) is justified by recognizing that the former represents a natural choice given that the stakeholders we engage with all operate in Sweden or in the neighbouring countries. The latter represents more than 53% of global economic losses due to natural disaster in 2022 [38]. We therefore expect mature and abundant research knowledge and experience coming from this part of the world. We do acknowledge that other parts of the world are exposed to climate risk but differences in building regulation and geographic conditions might make the comparison with Europe and North America less robust. We anticipate that our findings could be relevant to other regions, as it will provide evidence for the causal link between the different types of climate risks; real estate assets; and real estate value. For example, our mapping of the methodologies used to monetize the damage to real estate assets could also be applicable to other settings. Finally, our heatmap will show whether certain hazards are under- or over-researched, which could be insightful for further studies both within the focus geographies and elsewhere.

#### Study validity assessment

We acknowledge a potential for systematic error in our evidence synthesis, which could be due to a risk of bias in the primary studies we include in our map (internal validity) but also as due to including or excluding articles that are not fit for purpose (external validity) [39]. While we will not conduct a formal validity assessment (see, for example, [40]), we will include in our coding framework study design elements (such as the approach used to model the risk and account for damages), which will allow to get insight into the robustness of the sample. We will also discuss with our consortium the potential for systematic error in our map, and validate the findings from our systematic map with the stakeholders (MAVERIC consortium partners), to provide some quality assurance over the robustness of our map.

#### Data coding strategy

From the final list of retained papers, a dataset consisting of authors, year of publication, journal, DOI, abstract and keywords will be populated by the reviewing team. As the topic of climate risk and real estate valuation is new, our coding framework will develop iteratively [41]. Below, we detail which information we expect to extract at full text level. In addition, the authors will each read 10 papers from the retained corpus, fill in the anticipated coding framework, and make suggestions for additional data extraction. The authors will then meet to discuss the coding framework and finalise the additional categories. The coding framework will also be presented to the stakeholders (MAVERIC consortium partners) for approval in October 2023. Feedback from the stakeholders and changes to the coding framework will be documented in a systematic way. A further validation exercise will take place in the Spring of 2024 with the stakeholders, where some preliminary results will be presented. At that time, further changes to the coding framework will be discussed and finalized.

Our draft coding framework (Additional file 2) entails:

- **Climate risks considered**: what are the hazard events that are mentioned in the papers? This includes finding mentions to hazards such as coastal flooding, fluvial flooding, sea level rise, droughts, hurricanes, tornados, tsunamis, wildfires, heat waves and others. What transitional risks are covered?
- **Location of the study:** country, region or more specific geographical identification.
- **Approach used to model the risk and account for damages**: if an estimation of potential or actual damages from exposure to climate risks is presented, we classify the methodology used (see, for example, [42] Fig. 2) as well as the model used to estimate the impact on the real estate value [43, 44]. This could, for instance, take the form of an econometric approach, where a series of control variables are used to estimate the amount of damages, the dependent variable.
- **Elements of the valuation affected**: we are interested in understanding what elements of the valuation of real estate assets (reparation costs, adaptation costs, lowered rent opportunities, etc.) are affected the most by the exposure to natural hazards or due to transition changes.
- **Type of buildings considered**: we investigate whether particular focus is put on one type of real estate (residential, offices, retail, hotels, industrial, other).
- **Timeframe of the analysis**: if an impact on the future flow of revenues is modeled, what is the timeframe being considered in the study?
- **Recommendations**: suggestions for different types of actors related, for example, on the better integration of climate risk in real estate valuation model, on regulation, on mitigating the risks, etc.
- **Scope for further analysis**: does the publication suggest further topics that future research may explore?
- **Further relevant notes**: anything relevant that may not fall into the categories that we have defined before.

#### Study mapping and presentation

The coding framework (Additional file 2) will inform the mapping of our findings. To ensure that there is no double counting of findings, we will present the results from peer-reviewed publications and non-peer-reviewed papers separately. Firstly, the locations of the case studies will be mapped to identify those countries, among the economies identified in the inclusion criteria, that were subject to the largest attention by the research community. This will give the research team the possibility to understand to what extent the Swedish case has been over or underrepresented in the previous literature compared to other countries. Secondly, the geographical locations of the case studies will also be mapped against the list of natural hazards considered in the literature to generate a heatmap matrix. This will allow us to test if there is a tendency in some countries to focus on one or more natural hazards than others. It will also make it possible to assess which natural phenomena have been most frequently linked to climate risk for real estate assets. We expect those events such as tornados and hurricanes to be mostly investigated in the context of North America, where these are more common than in Europe. Such a mapping exercise will also help the research team identify those climate risks that characterize the European cases the most. A similar approach will be applied to the studies that focus on transitional climate risk, to understand the conditions, geographical, economic, political, that prompted the researchers to identify this risk as a relevant one for real estate assets. Finally, a review of the methodologies applied so far will provide directions for further research. This final presentation will be in text form, listing the methods used in research, and any shortcomings or recommendations provided in the literature on the method. The mapping will highlight the applicability of the methods in different settings.

## Supplementary Information

**Additional file 1: Additional material 1.** Findings from the search string. **Additional material 2.** Coding framework. **Additional material 3.** List of 50 relevant papers.

**Additional file 2:** Roses.

## Data Availability

The datasets generated and/or analyses during the current study are available from the corresponding author on reasonable request.
