# Supplementary material for: What evidence exists on the impact of climate change on real estate valuation? A systematic map protocol
Source: Environ Evid. 2023 Nov 18;12:24. doi: 10.1186/s13750-023-00317-y (PMC11378814; doi:10.1186/s13750-023-00317-y)
Supplement: Supplementary file 1 — Additional file 1: Additional material 1. Findings from the search string. Additional material 2. Coding framework. Additional material 3. List of 50 relevant papers. [file 13750_2023_317_MOESM1_ESM.docx]

# Additional files

## Additional file 1. Findings from the search string

Table 2. Overview results from our search

| **Results** | **Scopus** | **Web of Science** | **Overton** |
| --- | --- | --- | --- |
| Search string - **full** | ALL ( climat* AND risk* AND ( value OR economic OR financ* ) AND ( real AND estate OR building* ) ) AND ( LIMIT-TO ( DOCTYPE , "ar" ) ) AND ( LIMIT-TO ( LANGUAGE , "English" ) ) AND ( LIMIT-TO ( SRCTYPE , "j" ) ) AND ( LIMIT-TO ( PUBSTAGE , "final" ) ) | TS=(climat* AND risk* AND (value OR economic OR financ* ) AND ( (real AND estate) OR building* ))  Refined by: Languages (English); Document type (Article) | (climat* AND risk* AND (value OR economic OR financ* ) AND ( (real AND estate) OR building* )) |
| Full results | 21,484 | 1,467 | 411,044 |
| Search string – **time limit** | ALL ( climat* AND risk* AND ( value OR economic OR financ* ) AND ( real AND estate OR building* ) ) AND PUBYEAR > 2012 AND ( LIMIT-TO ( SRCTYPE,"j" ) ) AND ( LIMIT-TO ( PUBSTAGE,"final" ) ) AND ( LIMIT-TO ( DOCTYPE,"ar" ) ) AND ( LIMIT-TO ( LANGUAGE,"English" ) ) | TS=(climat* AND risk* AND (value OR economic OR financ* ) AND ( (real AND estate) OR building* )) AND PY=(2013-2023)  Refined by: Languages (English); Document type (Article) | (climat* AND risk* AND (value OR economic OR financ* ) AND ( (real AND estate) OR building* ))  Refined by: published in the selected years (2013-2023) |
| Articles 2014 – present (no geographic constraints) | 20,075 | 1,301 | 299,682 |
| Search string – **time and geography limits** | ALL ( climat* AND risk* AND ( value OR economic OR financ* ) AND ( real AND estate OR building* ) ) AND PUBYEAR > 2012 AND ( LIMIT-TO ( SRCTYPE , "j" ) ) AND ( LIMIT-TO ( PUBSTAGE , "final" ) ) AND ( LIMIT-TO ( DOCTYPE , "ar" ) ) AND ( LIMIT-TO ( LANGUAGE , "English" ) ) AND ( LIMIT-TO ( AFFILCOUNTRY , "United States" ) OR LIMIT-TO ( AFFILCOUNTRY , "United Kingdom" ) OR LIMIT-TO ( AFFILCOUNTRY , "Italy" ) OR LIMIT-TO ( AFFILCOUNTRY , "Germany" ) OR LIMIT-TO ( AFFILCOUNTRY , "Spain" ) OR LIMIT-TO ( AFFILCOUNTRY , "Canada" ) OR LIMIT-TO ( AFFILCOUNTRY , "Netherlands" ) OR LIMIT-TO ( AFFILCOUNTRY , "France" ) OR LIMIT-TO ( AFFILCOUNTRY , "Portugal" ) OR LIMIT-TO ( AFFILCOUNTRY , "Sweden" ) OR LIMIT-TO ( AFFILCOUNTRY , "Poland" ) OR LIMIT-TO ( AFFILCOUNTRY , "Switzerland" ) OR LIMIT-TO ( AFFILCOUNTRY , "Turkey" ) OR LIMIT-TO ( AFFILCOUNTRY , "Greece" ) OR LIMIT-TO ( AFFILCOUNTRY , "Denmark" ) OR LIMIT-TO ( AFFILCOUNTRY , "Finland" ) OR LIMIT-TO ( AFFILCOUNTRY , "Norway" ) OR LIMIT-TO ( AFFILCOUNTRY , "Austria" ) OR LIMIT-TO ( AFFILCOUNTRY , "Belgium" ) OR LIMIT-TO ( AFFILCOUNTRY , "Ireland" ) OR LIMIT-TO ( AFFILCOUNTRY , "Romania" ) OR LIMIT-TO ( AFFILCOUNTRY , "Czech Republic" ) OR LIMIT-TO ( AFFILCOUNTRY , "Lithuania" ) OR LIMIT-TO ( AFFILCOUNTRY , "Hungary" ) OR LIMIT-TO ( AFFILCOUNTRY , "Slovakia" ) OR LIMIT-TO ( AFFILCOUNTRY , "Serbia" ) OR LIMIT-TO ( AFFILCOUNTRY , "Cyprus" ) OR LIMIT-TO ( AFFILCOUNTRY , "Slovenia" ) OR LIMIT-TO ( AFFILCOUNTRY , "Croatia" ) OR LIMIT-TO ( AFFILCOUNTRY , "Estonia" ) OR LIMIT-TO ( AFFILCOUNTRY , "Ukraine" ) OR LIMIT-TO ( AFFILCOUNTRY , "Luxembourg" ) OR LIMIT-TO ( AFFILCOUNTRY , "Latvia" ) OR LIMIT-TO ( AFFILCOUNTRY , "Iceland" ) OR LIMIT-TO ( AFFILCOUNTRY , "Malta" ) OR LIMIT-TO ( AFFILCOUNTRY , "Bosnia and Herzegovina" ) OR LIMIT-TO ( AFFILCOUNTRY , "Bulgaria" ) OR LIMIT-TO ( AFFILCOUNTRY , "Georgia" ) OR LIMIT-TO ( AFFILCOUNTRY , "North Macedonia" ) OR LIMIT-TO ( AFFILCOUNTRY , "Albania" ) OR LIMIT-TO ( AFFILCOUNTRY , "Armenia" ) OR LIMIT-TO ( AFFILCOUNTRY , "Montenegro" ) OR LIMIT-TO ( AFFILCOUNTRY , "Belarus" ) OR LIMIT-TO ( AFFILCOUNTRY , "Moldova" ) OR LIMIT-TO ( AFFILCOUNTRY , "Andorra" ) ) | TS=(climat* AND risk* AND (value OR economic OR financ* ) AND ( (real AND estate) OR building* )) AND PY=(2013-2023)  Refined by: Languages (English); Document type (Article); Geographic limit | (climat* AND risk* AND (value OR economic OR financ* ) AND ( (real AND estate) OR building* ))  Refined by: published in the selected years (2013-2023) and “from Europe” OR “from North America” |
| Articles 2014-present and restricted to Europe and North America | 13,598 | 963 | 207,438 |

## Additional file 2. Coding framework

Table 3. Anticipated coding framework

| **Variable** | **Options** |
| --- | --- |
| Type of climate risk | Physical risk  Transition risk |
| Type of physical risk | Inspired by (23) and (24):   - Temperature-related chronic: changing temperature, heatstress, temperature variability, permafrost thawing - Temperature-related acute: heat wave, cold wave/frost, wildfire - Wind-related chronic: changing wind patterns - Wind-related acute: cyclone, hurricane, typhoon, storm (blizzards, dust and sandstorms), tornado - Water-related chronic: changing precipitation patterns and types; precipitation and/or hydrological variability, ocean acidification, saline intrusion, sea level rise, water stress - Water-related acute: drought, heavy precipitation (rain, hail, snow/ice), flood (coastal, fluvial, pluvial, ground water), glacial lake outburst - Solid mass-related chronic: coastal or soil erosion, soil degradation, solifluction - Solid mass-related acute: avalance, landslide, subsidence |
| Type of transitional risk | - Policy and legal risks - Liability risk - Technology risk - Market risk, and - Reputation risk |
| Location of the study | (name city, region and/country) |
| Approach used to model the risk | Inspired by (38), including:   - Integrated Assessment Model (IAM) - UKCCRA - WorldRiskIndex |
| Approach used to model the damage | Methodology used to assess the impact of the climate risk on the valuation, such as:   - P-spline generalized additive hedonic model (GAM) (43) - Monte Carlo simulation (44) - Depth-damage models |
| Elements of the valuation affected | Reparation costs  Adaptation costs  Lowered rent opportunities  Etc. |
| Type of buildings considered | Residential  Offices  Industrial  Retail  Hotels, and  Others |
| Timeframe of the analysis | Forecasted, future damage or actual, historial damage  (year of the forecasted value or actual damage) |
| Recommendations | Suggestions put forward in the paper related to, for example, the calculation methodology, regulation around climate risk and real estate valuation, mitigation of climate risk, etc. |
| Scope for further analysis | Research gaps mentioned in the paper |
| Further relevant notes | Any other relevant statement in the paper that could be useful but does not fall under the different categories of the coding framework. |

## Additional file 3. List of 50 relevant papers

Table 4. List of 50 relevant papers

| **Year** | **Authors** | **Title** | **Journal** |
| --- | --- | --- | --- |
| 2020 | Spanner, Maximilian M.; Wein, Julia | Carbon risk real estate monitor: making decarbonisation in the real estate sector measurable | Journal of European Real Estate Research |
| 2021 | Welker, Christoph; Röösli, Thomas; Bresch, David N. | Comparing an insurer's perspective on building damages with modelled damages from pan-European winter windstorm event sets: a case study from Zurich, Switzerland | Natural Hazards and Earth System Sciences |
| 2020 | Westcott, Mark; Ward, John; Surminski, Swenja; Sayers, Paul; Bresch, David N.; Claire, Bronwyn | Be Prepared: Exploring Future Climate-Related Risk for Residential and Commercial Real Estate Portfolios | The Journal of Alternative Investments |
| 2019 | Hirsch, Jens; Spanner, Maximilian; Bienert, Sven | The Carbon Risk Real Estate Monitor—Developing a Framework for Science-based Decarbonizing and Reducing Stranding Risks within the Commercial Real Estate Sector | Journal of Sustainable Real Estate |
| 2020 | Kellermann, Patric; Schröter, Kai; Thieken, Annegret H.; Haubrock, Sören-Nils; Kreibich, Heidi | The object-specific flood damage database HOWAS 21 | Natural Hazards and Earth System Sciences |
| 2018 | Ortega, Francesc; Taṣpınar, Süleyman | Rising sea levels and sinking property values: Hurricane Sandy and New York’s housing market | Journal of Urban Economics |
| 2016 | Votsis, Athanasios; Perrels, Adriaan | Housing Prices and the Public Disclosure of Flood Risk: A Difference-in-Differences Analysis in Finland | Journal of Real Estate Finance and Economics |
| 2018 | McAlpine, Steven A.; Porter, Jeremy R. | Estimating Recent Local Impacts of Sea-Level Rise on Current Real-Estate Losses: A Housing Market Case Study in Miami-Dade, Florida | Population Research and Policy Review |
| 2015 | Putra, H.C.; Zhang, H.; Andrews, C. | Modeling real estate market responses to climate change in the coastal zone | JASSS |
| 2021 | Fisher, J.D.; Rutledge, S.R. | The impact of Hurricanes on the value of commercial real estate | Business Economics |
| 2020 | Kim, S.K.; Peiser, R.B. | The implication of the increase in storm frequency and intensity to coastal housing markets | Journal of Flood Risk Management |
| 2020 | Baldauf, Markus; Garlappi, Lorenzo; Yannelis, Constantine | Does Climate Change Affect Real Estate Prices? Only If You Believe In It | The Review of Financial Studies |
| 2021 | Hino, Miyuki; Burke, Marshall | The effect of information about climate risk on property values | Proceedings of the National Academy of Sciences of the United States of America |
| 2019 | Bernstein, Asaf; Gustafson, Matthew T.; Lewis, Ryan | Disaster on the horizon: The price effect of sea level rise | Journal of Financial Economics |
| 2018 | McCoy, Shawn J.; Walsh, Randall P. | Wildfire risk, salience & housing demand | Journal of Environmental Economics and Management |
| 2020 | Murfin, Justin; Spiegel, Matthew | Is the Risk of Sea Level Rise Capitalized in Residential Real Estate? | The Review of Financial Studies |
| 2022 | Kim, Seung Kyum; Hammitt, James K. | Hurricane risk perceptions and housing market responses: the pricing effects of risk-perception factors and hurricane characteristics | Natural Hazards |
| 2023 | Gourevitch, Jesse D.; Kousky, Carolyn; Liao, Yanjun (Penny); Nolte, Christoph; Pollack, Adam B.; Porter, Jeremy R.; Weill, Joakim A. | Unpriced climate risk and the potential consequences of overvaluation in US housing markets | Nature Climate Change |
| 2015 | Cupal, Martin | Flood Risk as a Price-setting Factor in the Market Value of Real Property | Procedia Economics and Finance |
| 2015 | Hirsch, Jens; Braun, Thomas; Bienert, Sven | Assessment of climatic risks for real estate | Property Management |
| 2020 | Paprotny, Dominik; Kreibich, Heidi; Morales-Nápoles, Oswaldo; Terefenko, Paweł; Schröter, Kai | Estimating exposure of residential assets to natural hazards in Europe using open data | Natural Hazards and Earth System Sciences |
| 2020 | Paprotny, Dominik; Kreibich, Heidi; Morales-Nápoles, Oswaldo; Castellarin, Attilio; Carisi, Francesca; Schröter, Kai | Exposure and vulnerability estimation for modelling flood losses to commercial assets in Europe | Science of The Total Environment |
| 2019 | Semenenko, Igor; Yoo, Junwook | Climate Change and Real Estate Prices | International Journal of Economics and Finance |
| 2016 | Meldrum, James R. | Floodplain Price Impacts by Property Type in Boulder County, Colorado: Condominiums Versus Standalone Properties | Environmental and Resource Economics |
| 2015 | Atreya, Ajita; Ferreira, Susana | Seeing is Believing? Evidence from Property Prices in Inundated Areas | Risk Analysis |
| 2020 | Donadelli, M.; Jüppner, M.; Paradiso, A.; Ghisletti, M. | Tornado activity, house prices, and stock returns | The North American Journal of Economics and Finance |
| 2017 | Saginor, Jesse; Ge, Yue | Do hurricanes matter? A case study of the residential real estate market in Brunswick County, North Carolina | International Journal of Housing Markets and Analysis |
| 2018 | Kiel, Katherine A.; Matheson, Victor A. | The effect of natural disasters on housing prices: An examination of the Fourmile Canyon fire | Journal of Forest Economics |
| 2022 | Wang, Haoying | Boundary discontinuity, unobserved spatial heterogeneity, and flood risk analysis: application to a rural housing market | GeoJournal |
| 2023 | Mutlu, Asli; Roy, Debraj; Filatova, Tatiana | Capitalized value of evolving flood risks discount and nature-based solution premiums on property prices | Ecological Economics |
| 2019 | Pilla, Francesco; Gharbia, Salem S.; Lyons, Ronan | How do households perceive flood-risk? The impact of flooding on the cost of accommodation in Dublin, Ireland | The Science of the Total Environment |
| 2019 | Shr, Yau-Huo (Jimmy); Zipp, Katherine Y. | The Aftermath of Flood Zone Remapping: The Asymmetric Impact of Flood Maps on Housing Prices | Land Economics |
| 2020 | Hennighausen, Hannah; Suter, Jordan | Flood Risk Perception in the Housing Market and the Impact of a Major Flood Event | Land Economics |
| 2019 | Beltrán, Allan; Maddison, David; Elliott, Robert | The impact of flooding on property prices: A repeat-sales approach | Journal of Environmental Economics and Management |
| 2023 | Livy, Mitchell R. | Assessing the housing price capitalization of non-destructive flooding events | Research in Economics |
| 2019 | Zhang, Lei; Leonard, Tammy | Flood Hazards Impact on Neighborhood House Prices | The Journal of Real Estate Finance and Economics |
| 2022 | Pollack, Adam B.; Kaufmann, Robert K. | Increasing storm risk, structural defense, and house prices in the Florida Keys | Ecological Economics |
| 2022 | Miller, Ryan G.; Pinter, Nicholas | Flood risk and residential real-estate prices: Evidence from three US counties | Journal of Flood Risk Management |
| 2022 | Shi, Longzhong; Chen, Bo; Chen, Xuan; Chen, Zhuo | Assessing the impact of wildfires on property values in wildland-urban intermix and interface in Colorado: A hedonic approach | Journal of Environmental Management |
| 2013 | Hansen, Winslow D.; Naughton, Helen T. | The effects of a spruce bark beetle outbreak and wildfires on property values in the wildland–urban interface of south-central Alaska, USA | Ecological Economics |
| 2014 | Mueller, Julie M.; Loomis, John B. | Does the estimated impact of wildfires vary with the housing price distribution? A quantile regression approach | Land Use Policy |
| 2019 | Atreya, Ajita; Czajkowski, Jeffrey | Graduated Flood Risks and Property Prices in Galveston County | Real Estate Economics |
| 2016 | Zhang, Lei | Flood hazards impact on neighborhood house prices: A spatial quantile regression analysis | Regional Science and Urban Economics |
| 2021 | Cohen, Jeffrey P.; Barr, Jason; Kim, Eon | Storm surges, informational shocks, and the price of urban real estate: An application to the case of Hurricane Sandy | Regional Science and Urban Economics |
| 2022 | Bakkensen, Laura A; Barrage, Lint | Going Underwater? Flood Risk Belief Heterogeneity and Coastal Home Price Dynamics | The Review of Financial Studies |
| 2013 | Bin, Okmyung; Landry, Craig E. | Changes in implicit flood risk premiums: Empirical evidence from the housing market | Journal of Environmental Economics and Management |
| 2023 | Graff Zivin, Joshua; Liao, Yanjun; Panassié, Yann | How hurricanes sweep up housing markets: Evidence from Florida | Journal of Environmental Economics and Management |
| 2018 | Mueller, Julie M.; Lima, Ryan E.; Springer, Abraham E.; Schiefer, Erik | Using Matching Methods to Estimate Impacts of Wildfire and Postwildfire Flooding on House Prices | Water Resources Research |
| 2020 | Boustan, Leah Platt; Kahn, Matthew E.; Rhode, Paul W.; Yanguas, Maria Lucia | The effect of natural disasters on economic activity in US counties: A century of data | Journal of Urban Economics |
| 2022 | Dogan, Can; Hattapoglu, Mustafa; Hoxha, Indrit | Short-run effects of hurricanes on the housing markets: evidence from Florida | International Journal of Housing Markets and Analysis |
