# Supplementary material for: What evidence exists on the impact of climate change on real estate valuation? A systematic map protocol
Source: Environ Evid. 2023 Nov 18;12:24. doi: 10.1186/s13750-023-00317-y (PMC11378814; doi:10.1186/s13750-023-00317-y)
Supplement: Supplementary file 2 — Additional file 2: Roses. [file 13750_2023_317_MOESM2_ESM.pdf]

num	Section / sub-section	Topic	Description	Further explanation	Checklist/Meta-data	Author response	Comments
1	Title	Title	systematic map update protocol...".	question.	Meta-data	protocol	
2	Type of review	Type of review	Select one of the following types of review: systematic map, systematic map update, systematic map amendment	updates [2]	Meta-data	systematic map	
3	Authors contacts	Authors contacts	The full names, institutional addresses, and email addresses for all authors must be provided.		Checklist	Yes	
4	Abstract	Structured summary	including the review question; 2) Methods, how the review will be conducted and the outputs that are expected		Checklist	Yes	
5	Background	Background	necessary and what it aims to contribute to the field.	the intervention or exposure to the outcome.	Checklist	Yes	
6	Stakeholder engagement	Stakeholder engagement	described and explained (using a broad definition of 'stakeholder', including e.g. researchers, funders and other decision-		Checklist	Yes	
7	Objective of the review	Objective	Describe the primary question and secondary questions (when applicable).	questions are usually linked to sources of heterogeneity (effect	Checklist	Yes	
8		Definitions of the question components	outcome(s).	For other question types see [4,5]	Meta-data	structure	
	Methods						
9	Searches	Search strategy		Details regarding search strategy testing should be provided.	Checklist	Yes	
10		Search string	Provide Boolean-style full search string and state the platform for which the string is formatted (e.g. Web of Science format)		Meta-data	**Scopus:** ALL (climat* AND risk* AND (value OR economic OR financ*) AND (real AND estate OR building*)) AND (LIMIT-TO (DOCTYPE , "ar")) AND (LIMIT-TO (PUBYEAR , 2014) OR LIMIT-TO (PUBYEAR , 2015) OR LIMIT-TO (PUBYEAR , 2016) OR LIMIT-TO (PUBYEAR , 2017) OR LIMIT-TO (PUBYEAR , 2018) OR LIMIT-TO (PUBYEAR , 2019) OR LIMIT-TO (PUBYEAR , 2020) OR LIMIT-TO (PUBYEAR , 2021) OR LIMIT-TO (PUBYEAR , 2022) OR LIMIT-TO (PUBYEAR , 2023)) AND (LIMIT-TO (LANGUAGE , "English")) AND (LIMIT-TO (SRCTYPE , "j")) AND (LIMIT-TO (PUBSTAGE , "final")) **Web Of Science**: ALL=(climat* AND risk* AND (value OR economic OR financ*) AND (real AND estate OR building*)) and 2023 or 2022 or 2021 or 2020 or 2019 or 2018 or 2017 or 2016 or 2015 or 2014 (Publication Years) and Article (Document Types) and English (Languages)	
11		Languages – bibliographic databases	List languages to be used in bibliographic database searches.		Meta-data	English	
12		Languages – grey literature	List languages to be used in organizational websites searches and web-based search engines.		Meta-data	Not applicable	
13		Bibliographic databases	Provide the number of bibliographic databases to be searched.		Meta-data	2	
14		Web – based search engines	Provide the number of web – based search engines to be searched.		Meta-data	0	
15		Organisational websites	Provide the number of organisational websites to be searched.		Meta-data	0	
16		Estimating the comprehensiveness of the search	Describe the process by which the comprehensiveness of the search strategy was assessed (i.e. list of benchmark articles).		Checklist	Yes	
17		Search update	Describe any plans to update the searches during the conduct of the review.	performed more than two years prior to review completion.	Checklist	No	
18	inclusion criteria	Screening strategy	Describe the methodology for screening articles/studies for relevance/eligibility.		Checklist	Yes	
19		Consistency checking	undertaken and estimated proportion of articles/studies that will be screened and checked for consistency by two or more		Checklist	Yes	
20		Inclusion criteria	question key elements (e.g. relevant subject(s), intervention(s)/exposure(s), comparator(s), outcomes, study design(s)) and		Checklist	Yes	
21		Reasons for exclusion	State that you will provide a list of articles excluded at full text with reasons for exclusion.		Checklist	Yes	
22	Critical appraisal	Critical appraisal strategy	and the evidence base as a whole).	Optional	Checklist	No	
23		Critical appraisal used in synthesis	Describe how the information from critical appraisal will be used in synthesis.	Optional	Checklist	No	
24		Consistency checking	Describe how repeatability of critical appraisal of study validity will be tested.	Optional	Checklist	No	
25	Data extraction	Meta-data extraction and coding strategy	piloted), list if variables to be extracted as meta-data and those that will be coded).		Checklist	Yes	
26	presentation	Narrative synthesis strategy	(including SM database) and figures.	of their findings) must be avoided. May include a summary of the	Checklist	Yes	
27		Knowledge gap and cluster identification strategy	subtopics that warrant further primary research) and knowledge clusters (well-represented subtopics that are amenable to		Checklist	Yes	
28		Demonstrating procedural independence	regarding inclusion or critical appraisal of their own work.	review should be prevented from unduly influencing inclusion decisions,	Checklist	Yes	
29	Declarations	Competing interests	Describe of any financial or non-financial competing interests that the review authors may have.		Checklist	Yes	

renc

[1] James, K.L., Randall, N.P. and Haddaway, N.R., 2016. A methodology for systematic mapping in environmental sciences. Environmental Evidence, 5(1), p.7.

[2] Bayliss, H.R., Haddaway, N.R., Eales, J., Frampton, G.K. and James, K.L., 2016. Updating and amending systematic reviews and systematic maps in environmental management. Environmental Evidence, 5(1), p.20.

[3] Haddaway, N.R., Kohl, C., da Silva, N.R., Schiemann, J., Spök, A., Stewart, R., Sweet, J.B. and Wilhelm, R., 2017. A framework for stakeholder engagement during systematic reviews and maps in environmental management. *Environmental Evidence* , *6* (1), p.11.

[4] Collaboration for Environmental Evidence. 2018. Guidelines and Standards for Evidence synthesis in Environmental Management. Version 5.0. www.environmentalevidence.org/information-for-authors.

[5] Leeds Institute of Health Sciences. https://medhealth.leeds.ac.uk/info/639/information_specialists/1500/search_concept_tools. Accessed 12/11/2017.
